# Systematic comparison of local approaches for isotopically nonstationary metabolic flux analysis

**DOI:** 10.3389/fpls.2023.1178239

**Published:** 2023-06-06

**Authors:** Sebastian Huß, Zoran Nikoloski

**Affiliations:** ^1^ Systems Biology and Mathematical Modelling Group, Max Planck Institute of Molecular Plant Physiology, Potsdam, Germany; ^2^ Bioinformatics, Institute of Biochemistry and Biology, University of Potsdam, Potsdam, Germany

**Keywords:** isotopically nonstationary, metabolic flux analysis, local, comparison, flux estimation

## Abstract

Quantification of reaction fluxes of metabolic networks can help us understand how the integration of different metabolic pathways determine cellular functions. Yet, intracellular fluxes cannot be measured directly but are estimated with metabolic flux analysis (MFA) that relies on the patterns of isotope labeling of metabolites in the network. For metabolic systems, typical for plants, where all potentially labeled atoms effectively have only one source atom pool, only isotopically nonstationary MFA can provide information about intracellular fluxes. There are several global approaches that implement MFA for an entire metabolic network and estimate, at once, a steady-state flux distribution for all reactions with identifiable fluxes in the network. In contrast, local approaches deal with estimation of fluxes for a subset of reactions, with smaller data demand for flux estimation. Here we present a systematic comparative review and benchmarking of the existing local approaches for isotopically nonstationary MFA. The comparison is conducted with respect to the required data and underlying computational problems solved on a synthetic network example. Furthermore, we benchmark the performance of these approaches in estimating fluxes for a subset of reactions using data obtained from the simulation of nitrogen fluxes in the *Arabidopsis thaliana* core metabolism. The findings pinpoint practical aspects that need to be considered when applying local approaches for flux estimation in large-scale plant metabolic networks.

## 1 Introduction

Fluxes of biochemical reactions are the results of transcriptional, translational, and post-translational processes (Stitt et al., 2010). They affect and determine how and with which efficiency cellular resources are used, ultimately resulting in an observed cellular phenotype. As a consequence, quantifying metabolic fluxes is important to uncover the functionality of metabolic networks and provides the basis for identifying promising targets for metabolic engineering (Stephanopoulos, 1999; Segrè et al., 2002; Lewis et al., 2010; Razaghi-Moghadam and Nikoloski, 2021; Treves et al., 2022)

Intracellular reaction fluxes cannot be measured directly and, instead, are estimated by applying computational methods that integrate data from labeling experiments into a metabolic model. Stoichiometric models provide a mathematical description of biochemical reactions; together with assumed optimality principles and physiological constraints (e.g. maximized growth rate and steady state, respectively, as in flux balance analysis (FBA) (Orth et al., 2010)), such models are integral part of the constraint-based modeling framework (Bordbar et al., 2014) that allows prediction of steady-state flux distributions. However, estimation of fluxes by integration of data into metabolic models, as performed in metabolic flux analysis (MFA) (Antoniewicz, 2018; Basler et al., 2018), in addition to the mathematical description of reactions, requires an accurate mapping of the atom transitions taking place in the metabolic reactions. This does not only allow the integration of data gained from isotope tracer feeding experiments, but also leads to an increased precision of the flux estimates (Leighty and Antoniewicz, 2013).

MFA has two variants with respect to whether data from isotopically stationary or nonstationary state are used (Basler et al., 2018). Isotopically stationary state provides a snapshot of the system at which the incorporation of the label has reached stationarity. In contrast, experiments at isotopically nonstationary state imply performing time-resolved measurements that provide temporal data on the incorporation and redistribution of the label in the network. A wide range of biological systems has been studied using stationary ^13^C-MFA, from *Escherichia coli* metabolism (Sandberg et al., 2016; Fischer and Sauer, 2003) to central carbon metabolism in heterotrophic plant tissues (Williams et al., 2008; Masakapalli et al., 2010) and cancer cell lines (Antoniewicz, 2018; Liang et al., 2021).

For labeling experiments with plants grown under mixo- or hetero-trophic growth, with partially labelled C atoms in glucose as an (additional) carbon source the isotopically stationary state can be informative for flux estimation. This is the case since at the isotopically stationary state, for this labeling set-up, not all C atoms of all metabolites will be fully labelled, and classical approaches from MFA can be employed for flux estimation. For labeling experiments with plants grown autotrophically, CO_2_ is the only carbon source; as a result, labeling its single carbon will lead to an isotopically stationary state in which all metabolites are fully labeled. As a result, measuring the isotopic stationary state will have equivalent information about fluxes as measurements of total pool sizes. Similar issue arises for nitrogen labeling experiments with ammonium and nitrate, not only in plants but also in model bacteria, like *E. coli* (Yuan et al., 2006). The second variant of MFA, referred to as INST-MFA, considers data from an isotopically nonstationary state and was developed to specifically address the estimation of fluxes for such scenarios.

The integration of labeling patterns into metabolic models in INST-MFA can be performed on a global level, using all reactions in a given metabolic model and imposing constraints on exchange fluxes. This global INST-MFA approach leads to a computationally difficult problem for which widely applied toolboxes exist (e.g. INCA (Young, 2014)) alongside other implementations (e.g. using the general algebraic modeling system (GAMS) (Lugar and Sriram, 2022) or MATLAB (Gopalakrishnan et al., 2018)). The mentioned implementations rely on the concept of elementary metabolite units (Antoniewicz et al., 2007) to simulate all labeling patterns for a steady-state flux distribution in a given metabolic network. However, application of the global INST-MFA may be challenging for several reasons. For instance, a large-scale metabolic network usually contains metabolites that have very different labeling time scales determined by the ratio of the metabolite pool size and the sum of the flux values of reactions involving this metabolite. The global INST-MFA must then be able to handle the different time scales arising in large-scale metabolic models. Further, the sheer number of metabolites and reaction contained in a large-scale metabolic network requires solving of a large inverse problem underlying the estimation of a steady-state flux distribution that yields the observed labeling patterns. Such inverse problems often lead to numerical instabilities. Finally, the estimation of steady-state fluxes in a given large-scale metabolic network is further aggravated by the limited number of metabolites for which isotopic labeling patterns are gathered in labeling experiments. Nevertheless, the global approaches can be used to obtain estimates of all identifiable fluxes in the network at once, allowing genome-scale insights in the flux patterns over multiple experimental scenarios.

In contrast, local approaches for INST-MFA, that only estimate the flux of a specific reaction or a subset of reactions in a sub-network, circumvent these issues due to the much smaller size of the resulting computational problems. Therefore, local INST-MFA approaches offer an alternative if fluxes of only few reactions are of interest. In this analysis review, three such local approaches for INST-MFA, namely: kinetic flux profiling (KFP) (Yuan et al., 2006), non-stationary metabolic flux ratio analysis (NSMFRA) (Hörl et al., 2013), and ScalaFlux (Millard et al., 2020), are compared and contrasted in two flux estimation scenarios. In the first scenario, we used a simple synthetic example network, which is part of the IsoSim repository (https://github.com/MetaSys-LISBP/IsoSim). This existing test case was further modified to determine the effect of the number of simulated measurements and different simulated measurement errors. In the second scenario, these local INST-MFA approaches were tested on the nitrogen flux through the metabolic network model AraCore of *A. thaliana* central metabolism (Arnold and Nikoloski, 2014). The review points at important consideration for planning of labeling experiments for application of local approaches for INST-MFA, particularly of interest to plant biology research.

## 2 Input and output of local INST-MFA approaches

All three local approaches for INST-MFA require as input the structural information of the sub-network containing the reaction whose fluxes are to be estimated. This structural information consists of the reactions in the network along with the involved metabolites and their stoichiometry. In addition, NSMFRA and ScalaFlux also require the mappings of atom transitions for the considered reactions. For instance, for ^13^C or ^15^N labeling experiments, the transition map of all carbon or nitrogen atoms must be provided alongside with the subnetwork.

The input for all approaches also includes the experimental data of the isotopomer distribution for the metabolites involved in the sub-network. In experiments which use mass spectrometry to identify mass isotopomer distributions (MIDs) of the considered metabolites, usually the relative abundance of isotopomers with the same mass, called cumomers, is measured (Wiechert et al., 1999). The class of a cumomer is denoted by M+x, where x represents the number of occurrences of the heavier isotope. We note that KFP makes use of only the unlabeled (M+0) isotopomer fraction; in contrast, ScalaFlux and NSMFRA consider all isotopomer fractions.

**Figure 1:**
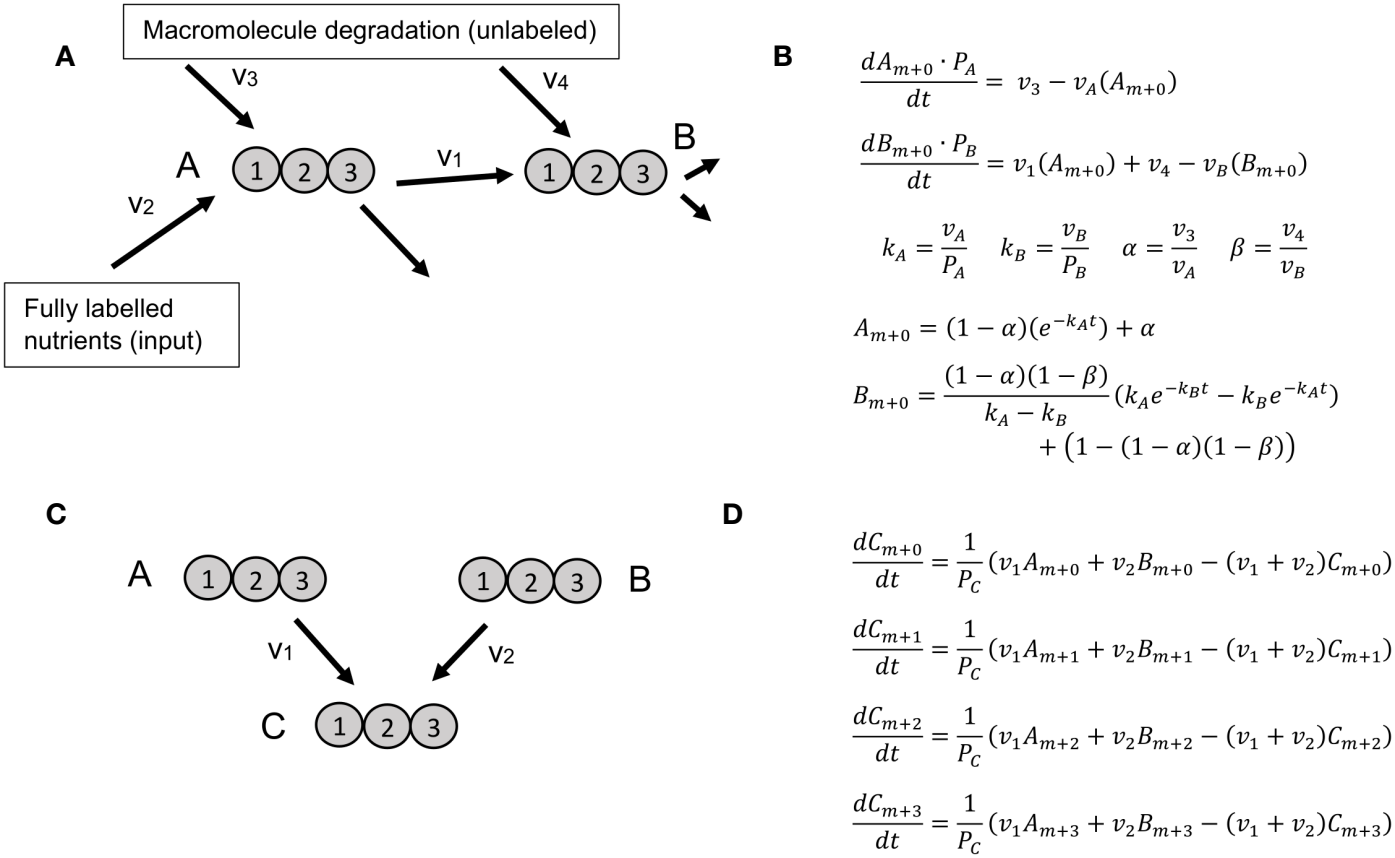
Reaction motifs and resulting ODEs for KFP and NSMFRA. **(A)** Network motif for which KFP is applicable includes a metabolite, *B*, that is a product of a reaction using the labeled metabolite *A* as substrate as well as a product of macromolecule degradation. Labeled atoms are introduced only from metabolite *A*, that is a product of fully labeled nutrients and unlabeled macromolecule degradation. **(B)** A system of ODEs for the motif on panel **(A)**, along with its analytical solution. **(C)** The simplest network motif for NSMFRA, consisting of two unimolecular reactions converging at one metabolite. **(D)** A system of ODEs for the MIDs. The system of ODEs allows the estimation of the parameters v_1_, v_2_, and P_Z_.

The output of KFP is limited to estimate the total flux through one metabolite, instead of individual reactions. Both KFP and NSMFRA require data on absolute metabolite concentration data only in the case when absolute fluxes are of interest; in absence of such data, these approaches can still estimate relative fluxes, i.e. the fractional turnover of metabolites. We note that NSMFRA is the only of the three compared approaches that uses simulated labeling data based on Hill kinetics for metabolites without measured isotopomers. However, the applicability of NSMFRA is limited to specific metabolites in the metabolic network, where pathways converge. The approach estimates the relative local fluxes at these nodes. In contrast, ScalaFlux is designed to estimate fluxes for any reaction or a subnetwork for which sufficient labeling data are available. The principle of KFP can also be adapted to many more network motifs than the simple one, shown in **Figure 1A**, as was done in eKFP (Szecowka et al., 2013).

All three approaches then solve the ODEs describing the change of the MID fractions with the reaction fluxes as parameters, optimized to fit the measurements or specified analytic functions (see below for details). In the case of ScalaFlux/IsoSim, the ODEs to solve are constructed from the network structure in an automated fashion. In contrast, for KFP and NSMFRA, setting up the ODEs must be performed manually, since no automated implementation is available. In the following, we provide a brief overview of the mathematical formulation and computational details of the three existing INST-MFA approaches.

## 3 Mathematical formulation of local INST-MFA approaches

KFP relies on a system of ODEs for the unlabeled fraction of two metabolites, or which one is a product of a single reaction that uses a labeled substrate; in addition, some amount of recycling from other, unlabeled molecules is allowed; on (**Figure 1A**). The system of ODEs can be solved analytically yielding the flux through the product metabolite (**Figure 1B**).

In NSMFRA, the ODEs are defined for two reactions that converge at one metabolite – a network motif particular to NSMFRA. This motif defines the ODE which is used to estimate the flux ratio of the two reactions. The two reactions can both be unimolecular (**Figure 1C**); in addition, the approach is applicable to the case where one of the reactions is either a cleavage or a condensation reaction. **Figure 1D** shows the equations for the MID fractions of the involved metabolites for two converging unimolecular reactions. As the molecules in the example have three atoms each, the MIDs are m+0, m+1, m+2 and m+3, the system of corresponding ODEs allows the estimation of the parameters v_1_, v_2_, and P_Z_.

**Figure 2:**
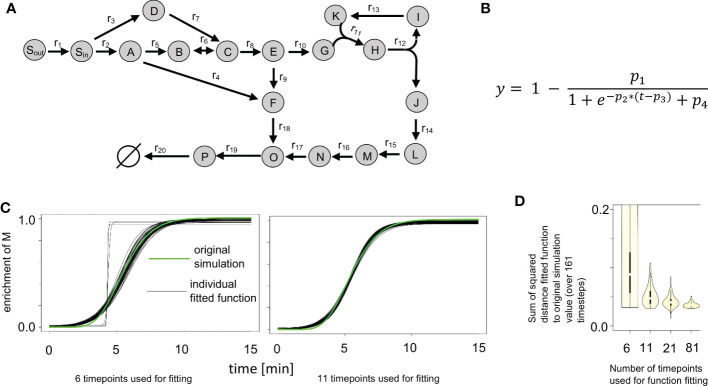
Impact of number of time points and added noise on labeling curve fit. **(A)** Synthetic network from ScalaFlux (Millard et al., 2020). **(B)** Logistic function used in fitting the enrichment data. **(C)** Plots of 100 fitted functions for the enrichment of M, obtained while labeling data with 6 and 11 time points were used, compared with the original simulation data. **(D)** Distribution of the sum of the squared errors between the fitted functions and the original simulation data. We used labeling data with 6, 11, 21 and 81 time points, each with 100 instances of randomly added noise. The usage of more time points expectedly leads to smaller variance in the fitted function.

**Figure 3:**
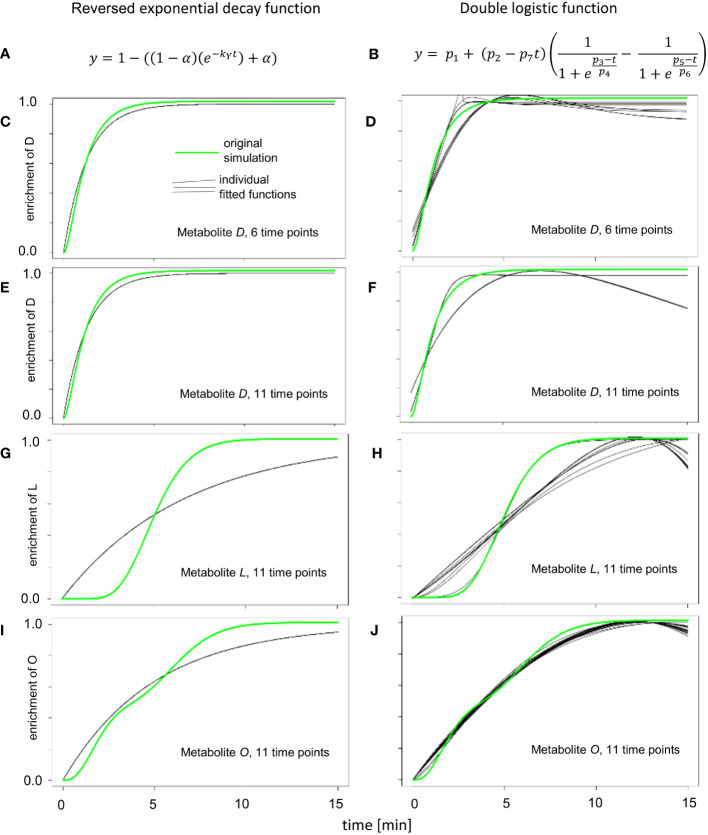
Enrichment curve fitting for reversed exponential decay and double logistic functions. **(A)** Reversed exponential decay function, as described for KFP (Yuan et al., 2008). **(B)** Double logistic function, that is a part of IsoSim/ScalaFlux (Millard et al., 2020). **(C, E, G, I)** The results of fitting 20 instances of simulated enrichment data with added random noise to the reversed exponential decay function. **(D, F, H, J)** The results of fitting 20 instances of simulated enrichment data with added random noise double logistic function.

ScalaFlux/IsoSim estimates the fluxes of reactions for a subsystem in a given metabolic network, where enrichment measurements are given for all input and output metabolites. This subsystem may contain only one reaction, but it can also be successively scaled to include the entire network. For the estimation, ScalaFlux/IsoSim follows a three-step approach. In the first step, the MIDs of all metabolites, which serve as input to the subsystem, are fitted to a logistic function (**Figure 2B**) and a double logistic function (**Figure 3B**). The selection of a (double) logistic function to fit the enrichment of label inputs in IsoSim is purely based on the observed goodness-of-fit. If the labeling dynamics of some metabolite is not described by the selected function, another, better suited function can be used. The fitting is performed using particle swarm optimization (Clerc 2006) multiple times for both functions employed. The fit with the smallest sum of squared distances between the time-resolved measurements and the simulated values of the fitted function is then chosen. In the second step, these functions serve now as part of the ODEs in the optimization problem to estimate the fluxes of the reactions in the subsystem. The ODEs are automatically created according to the network structure of the subsystem, with the flux values of the reactions as parameters. The system of ODEs allows to simulate the evolution of the MIDs of the considered metabolites. To estimate fluxes, nonlinear least square optimization is used to minimize the sum of squared distances of the time-resolved simulated and measured MID values. In a third step, a sensitivity analysis is conducted by repeating the second step a specified number of times with addition of noise according to the expected variance of MID measurements. The noise is added to the measurements of output metabolites, which are used as optimization targets. The resulting distribution of estimates defines the confidence intervals of the actual estimate.

## 4 Implementations of local INST-MFA approaches

ScalaFlux is the only of the compared local INST-MFA approaches that is available in a ready-to-use implementation. The implementation is available in the form of IsoSim (Millard et al., 2020), a software toolkit that is readily applicable to any combination of metabolic network and experimental data. In contrast, NSMFRA (Hörl et al., 2013) and KFP (Yuan et al., 2006) are only available as mathematical formulations. To compare the three approaches, we first implemented NSMFRA and KFP in R (https://github.com/sebahu/Local_INST-MFA), allowing us to apply them on the example network included in the IsoSim implementation.

## 5 Comparative analysis of performance for INST-MFA approaches

To compare the three approaches, we applied them with simulated data from two distinct networks: (1) the synthetic example network from ScalaFlux/IsoSim and (2) nitrogen fluxes in the AraCore model network with atom mappings obtained from Huß et al. (2022). The performance of the approaches was investigated with special consideration on the number of used measurement time points. We opted to examine the effect of this factor since measuring isotope labeling distributions is resource-intensive, and, thus, the number of time points is a limiting factor to obtain reliable flux estimation.

The simulation data for the synthetic example was sampled at 161 time points, specified by t_i_ = 10^(i-1)log^_10_^(16)/160^-1, 1 ≤ *i* ≤ 161, from a 15-minute-long simulation of the network. More specifically, the second time point was ~1 second after the start of the simulation and the second to last timepoint was ~16 seconds before the end of the simulation. The nitrogen labeling of the AraCore model network was sampled equidistantly at 81 time points for a 320-minute-long simulation. For the performance analysis, four subsets of these samples, containing 6, 11, 21 and 81 measurements, were used for each network.

## 6 Discrimination of input functions based on goodness of fit

KFP uses an exponential decay function to describe the decay of the unlabeled fraction of the input metabolite (*A_m+0_* in **Figure 1B**). For metabolites with only one atom that can be labeled, the enrichment of labeling equals one minus the unlabeled fraction of that metabolite. We denote the resulting function (**Figure 3A**) as reversed exponential decay function. This reversed exponential decay function and the input fitting functions of IsoSim were tested with simulated data from the synthetic network (**Figure 2A**). The synthetic network contains 17 metabolites with one atom and one metabolite, *H*, with two atoms. The input fitting was tested for all one-atom metabolites except S_out_ (since S_out_ was set to be fully labeled from the start). IsoSim fits the input labeling data for each metabolite to a logistic and a double logistic function, each multiple times with randomized starting values. The fit with the smallest distance to measurements is then chosen as the input function to the system of ODEs used for the flux estimation. In the case of the synthetic network and simulated flux distributions, in all but one case, a logistic function (**Figure 2B**) was selected as the best-fitting input function.

Next, we investigated the impact of the number of time points used as well as the impact of noise added to the measurements. We used labeling data with 6, 11, 21 and 81 time points as input, each with 100 instances of randomly added noise. This is equivalent to conducting a sensitivity analysis for the input fitting. **Figure 2C** shows the plots of 100 fitted functions for the enrichment of metabolite M in the synthetic network, resulting from the usage of labeling data with 6 and 11 time points. When only 6 time points were used, the variance of the fitted functions was expectedly the largest of the four scenarios (**Figure 2D**). The fitted functions were a poor match to the data, in some cases resulting in step-like fits (**Figure 2C**, gray lines). Already when using data from 11 time points, no step-like fits were observed in the fitting and the overall variance of the functions was already considerably smaller (**Figure 2D**). The plot shows that the use of more time points leads to even smaller variance in the fitted function, yet the most significant reduction was observed for the increase from 6 to 11 time points.

The simple reversed exponential decay function of KFP (**Figure 3A**) and the double logistic function from IsoSim (**Figure 3B**) resulted in worse fits for all but one atom pool (metabolite O, **Figure 3J**). The simple reversed exponential decay function of KFP is based on the metabolite being produced from fully labeled reactants (**Figure 1A**); as a result, it fails to describe the delayed start of isotope enrichment of atom pools (**Figure 3G**). For metabolites close to the input reaction of the network, which have only a small delay in the labeling, the simple reversed exponential decay function leads to fits nearly as good as those based on a logistic function, with a small distance to the measured data (**Figures 3C, E**). From its formulation, a double logistic function must be able to fit the data at least as good as a logistic function; this is the case since appropriate choice for the parameters of a double-logistic function render it a logistic function. Yet, as the results of fitting the double logistic function to 20 instances of simulation data with randomly added noise (**Figures 3D, F, H**) show, this is often not the case. This may be due to the increased number of parameters to fit in the double-logistic function, compared to four parameters for the logistic function, and can be explained by local optima in the fitting process and the effect of noise.

The metabolite *O* is at the junction where two metabolic paths with distinct delays of enrichment converge. These two paths end in the metabolites *F* and *N*, which show an enrichment following a logistic curve with differing parameters. This is the exact use case for fitting the double logistic function; as a result, the double logistic function has a better fit for metabolite *O* (**Figures 3I, J**).

## 7 Flux estimation for the synthetic network

Here, we aimed to estimate the flux of individual reactions, the most fine-grained local setting, since it also enabled us to compare the performance between approaches. First, we tested the approaches on simulated data from the synthetic network provided with the IsoSim implementation (https://github.com/MetaSys-LISBP/IsoSim). After careful evaluation of the source code, we decided to change the method of sensitivity analysis to be used with the data. IsoSim conducts a sensitivity analysis by repeating the second step of its process with randomized noise added to the measured (or in the test case, simulated) data, based on the standard deviation of the actual measurements (or expected standard deviation for the simulation). In doing so, however, the fitting of the input labeling data is not repeated. Since the fitting of input data is the first step in the process, with major impact on the resulting estimation, we decided to conduct our sensitivity analysis by repeating the whole process with noisy data. In addition, the default number of repetitions in IsoSim was rather low (fixed to 4), and we increased it to 100. This modified approach also allowed us to conduct comparative analyses with the other compared approaches. While these modifications increase the computation time, the steps are easy to parallelize.

Tests on an Intel Core-i5 8250U with all methods set to run on a single thread showed the following computation times: a single KFP-run for one reaction takes less than 0.1 seconds. The input fitting in IsoSim, which we also used for our implementation of the basic principle of NSMFRA, takes 40-60 seconds per metabolite. This includes multiple runs with randomized starting values for the logistic and the double logistic function, choosing the best run. The flux estimation for NSMFRA takes < 0.1sec per reaction. One estimation run in IsoSim for one reaction takes 1-4 sec.

**Figure 4:**
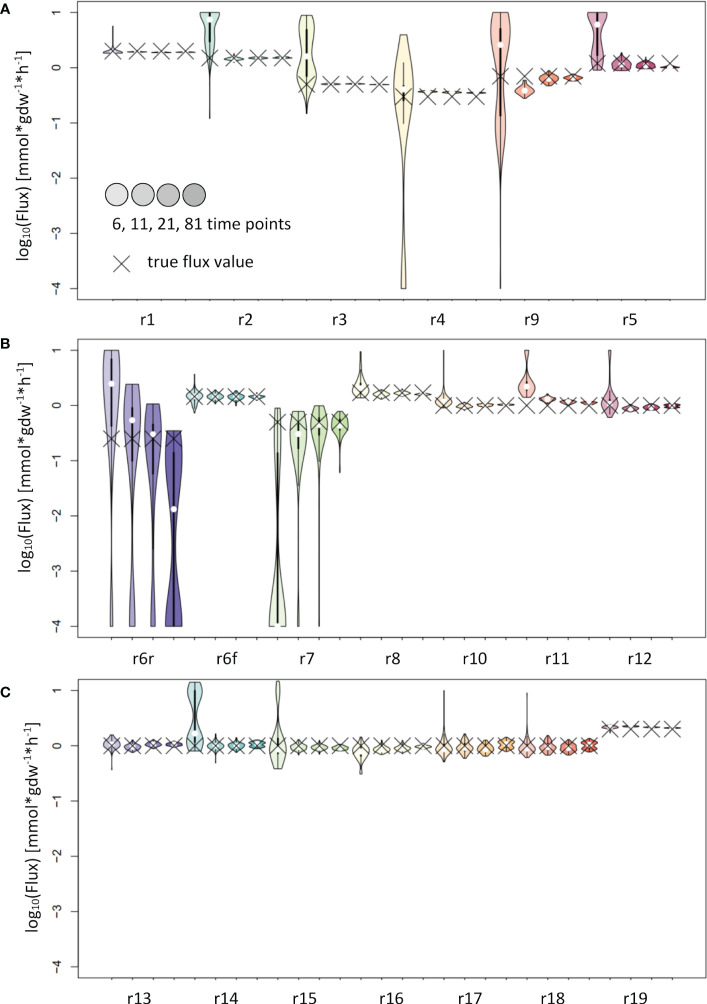
Flux estimates and sensitivity analysis of IsoSim for 6, 11, 21 and 81 time points from a synthetic network. **(A)** For reactions r1, r2, r3, r4, r5 and r9. **(B)** For reactions r6r, r6f, r7, r8, r10, r11 and r12. **(C)** For reactions r13, r14, r15, r16, r17, r18 and r19. In general, using eleven time points from the simulation of a synthetic network leads to flux estimates close to the true values, with small confidence intervals.

The analysis of the input fitting showed a major impact of the number of measurements on the result (**Figure 2**). To further evaluate the effect of this finding on flux estimation, we used labeling patterns with 6, 11, 21 and 81 time points as input. The results show good estimates for most reaction fluxes when labeling patterns with at least 11 time points were used as input (**Figure 4**). The noticeable exceptions include the flux of the backward reaction 6 and reaction 7 (**Figure 4B**); note that the product of reaction 7 serves as a reactant to the backward reaction 6. This kind of motif is best handled by including the surrounding network structure, e.g. by using the full coupling between the reactions 3 and 7 and the fact, that the sum of the flux of reactions 8 and backward 6 equals the sum of reactions 7 and forward 6 (**Figure 2A**). Instead of using the poor estimates for reactions 7 and backward 6, the estimates for reactions 3, 8 and forward 6 can be used to calculate reliable estimates for reactions 7 and backward 6.

**Figure 5:**
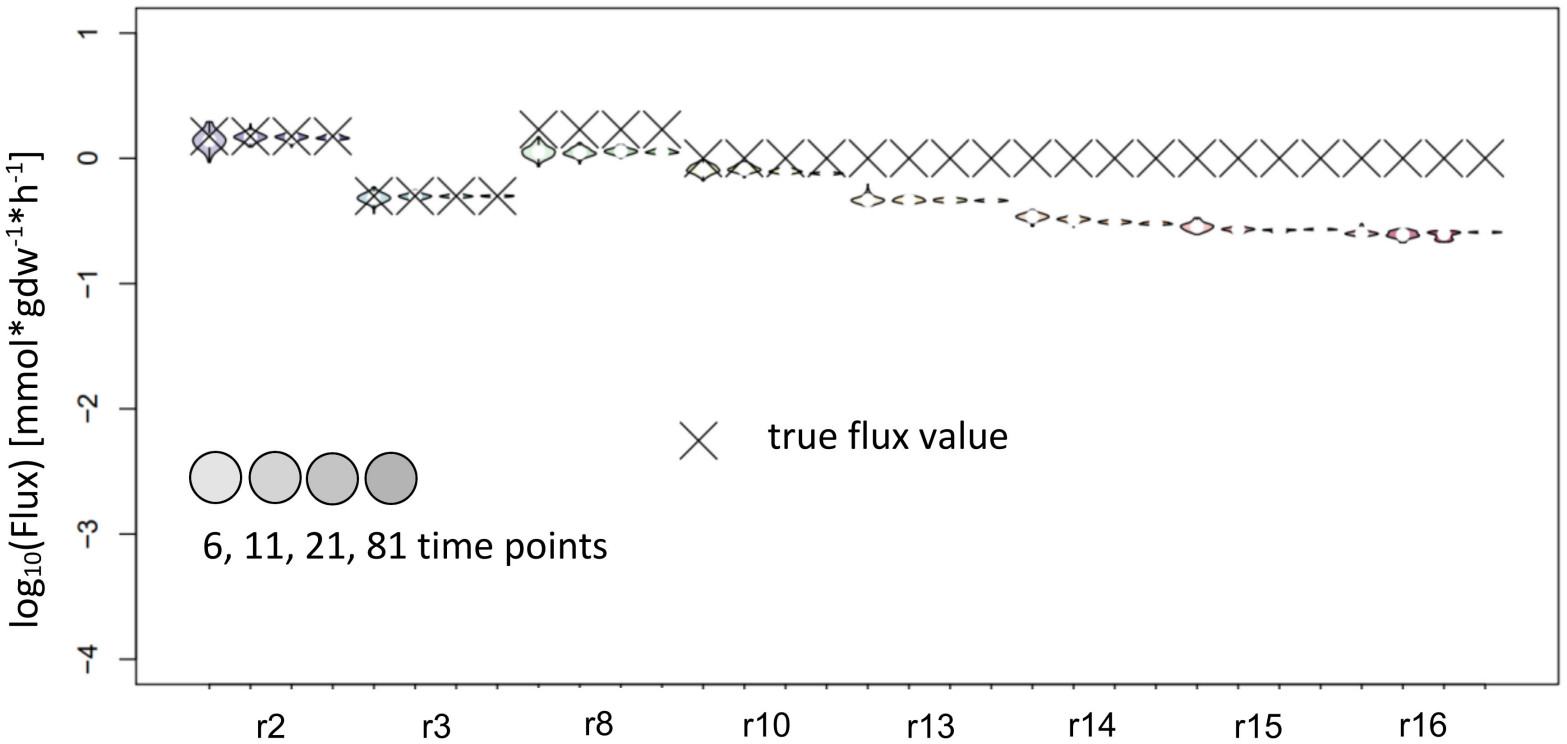
Flux estimates and sensitivity analysis of KFP for 6, 11, 21 and 81 time points for reactions of synthetic network. The confidence intervals of the estimated fluxes of individual reaction from IsoSim’s example network are very narrow, yet for many reactions, the true flux values are outside of these intervals.

As stated above, KFP estimates the total flux through a metabolite. If a metabolite is only produced by one reaction, the flux of this reaction equals the total flux through the metabolite and can be estimated by KFP. For our comparison, we chose such reaction/metabolite pairs for flux estimation with KFP. The input fitting derived from KFP worked well only on metabolites upstream in the network. Therefore, we focused the investigation on the effect of the labeling delay on the flux estimates. The results confirmed the applicability of KFP for reactions whose reactants are labeled shortly after the label is introduced (**Figure 5**). Interestingly, the confidence intervals for the respective reactions (r2 and r3) using six time points for the estimation were smaller than those obtained from IsoSim in the same setting. On the other hand, reactions further downstream in the network, with a delayed onset of labeling in their reactants, also have a small confidence interval; yet, the true flux value is not contained in these intervals. This is likely due to the systematic error in the input fitting to an reversed exponential decay curve (e.g. metabolite L, **Figure 3G**) and the assumptions of the KFP approach, detailed above. Such a fit also has a large sum of squared errors, showing that the method is not applicable for this reaction.

**Figure 6:**
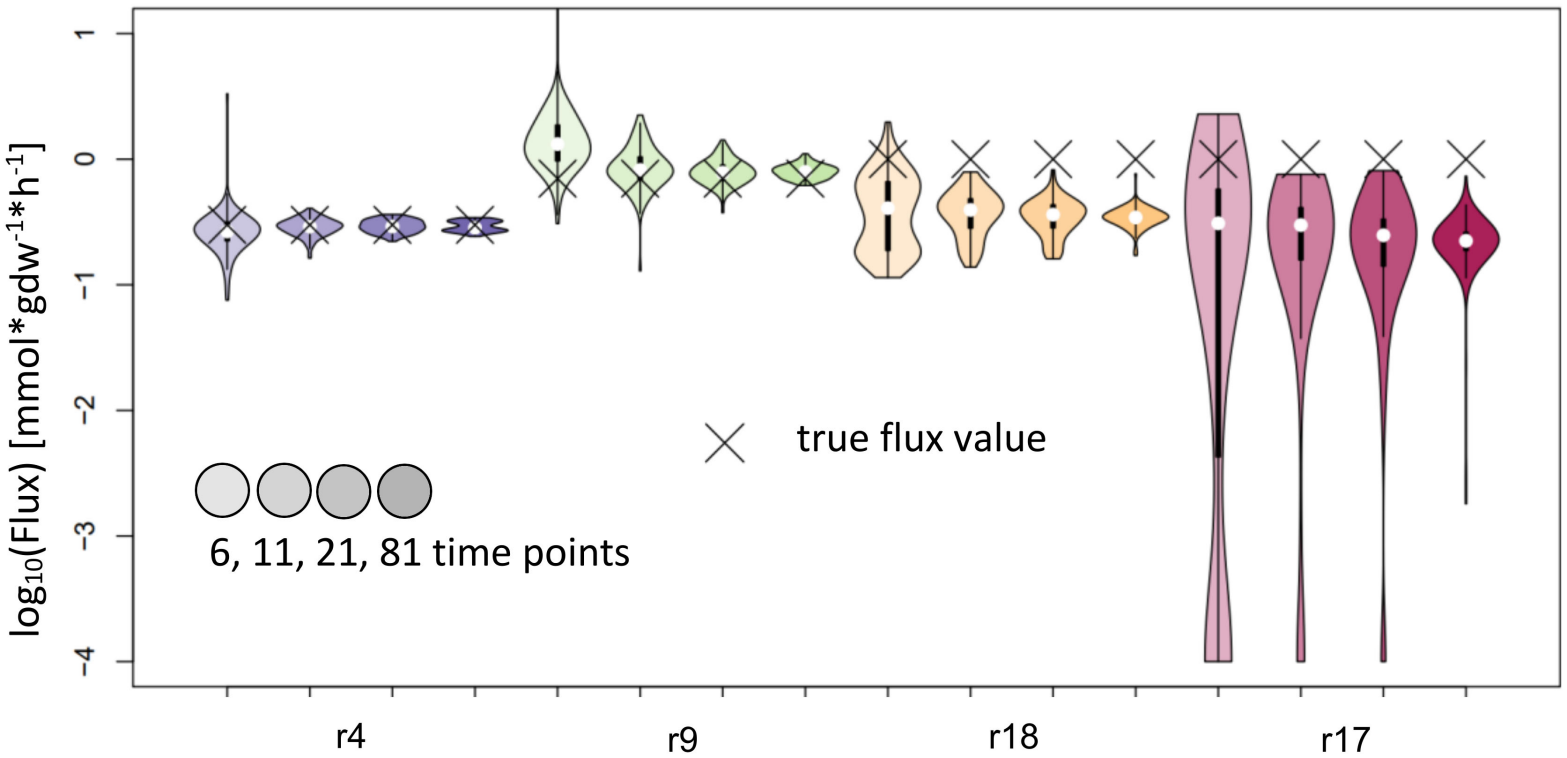
Flux estimates and sensitivity analysis of NSMFRA for 6, 11, 21 and 81 time points for reactions of synthetic network. The confidence intervals of the estimated fluxes for reactions 4 and 9 from the synthetic network are very small, yet for r18 and r17 the confidence intervals are bigger and the true flux values are outside of them.

NSMFRA is applicable to the reaction pair of reactions 4 and 9, and the to the reaction pair of reactions 17 and 18 in the synthetic network on **Figure 2A**. For NSMFRA, our implementation did not provide conclusive results (**Figure 6**). For instance, the reaction pair of reactions 4 and 9 could be estimated rather well, while for reactions 17 and 18 the method fails to provide reliable flux estimates (**Figure 6**).

## 8 Flux estimation for the AraCore model with labeled nitrogen input

To test the flux estimation for realistic metabolic networks, we employed the AraCore model (Arnold and Nikoloski, 2014) of *Arabidopsis thaliana* as a second model to simulate a labeling experiment and estimate fluxes. We simulated a nitrogen labeling experiment with ^15^N-labeled ammonium and nitrate as nitrogen sources to get insights in the challenges of characterizing nitrogen fluxes in this model plant. This simulation scenario is also a good match for the previously analyzed synthetic model, since in many reactions only one or two nitrogen atoms are involved.

The simulation was performed for the whole network labelled for 320 hours, with a flux distribution and metabolite pool sizes derived from a previous work (Huß et al., 2022). To further simplify the estimation process, the simulated labeling data were used in the compartmentalized form (in the cytoplasm, mitochondrion, and chloroplast). For the flux estimation, we focused on six reactions that satisfy the network motifs where the local INST-MFA approaches are applicable. Specifically, we chose Aspartate aminotransferase, Arogenate dehydratase, branched-chain amino acid aminotransferase and Methionine synthase since their products are not created by any other reactions in the model. As a result, the flux of these reactions equals the total flux through their respective products, and they can also be used to compare the estimates from the KFP approach. In addition, we selected the Threonine aldolase and Glycine hydroxymethyltransferase reactions as they converge into the same product, allowing the application of NSMFRA. In addition, these reactions were selected since they have an amino acid with one nitrogen atom as product, which is quite similar to the synthetic network tested.

As for the synthetic network, the fluxes of these reactions from the AraCore model were estimated with 6, 11, 21 and 81 time points. The sensitivity analysis was also conducted in the same fashion as for the synthetic model, using 100 repetitions with randomized noise added to the simulation data according to the expected standard derivation of actual experiments.

**Figure 7:**
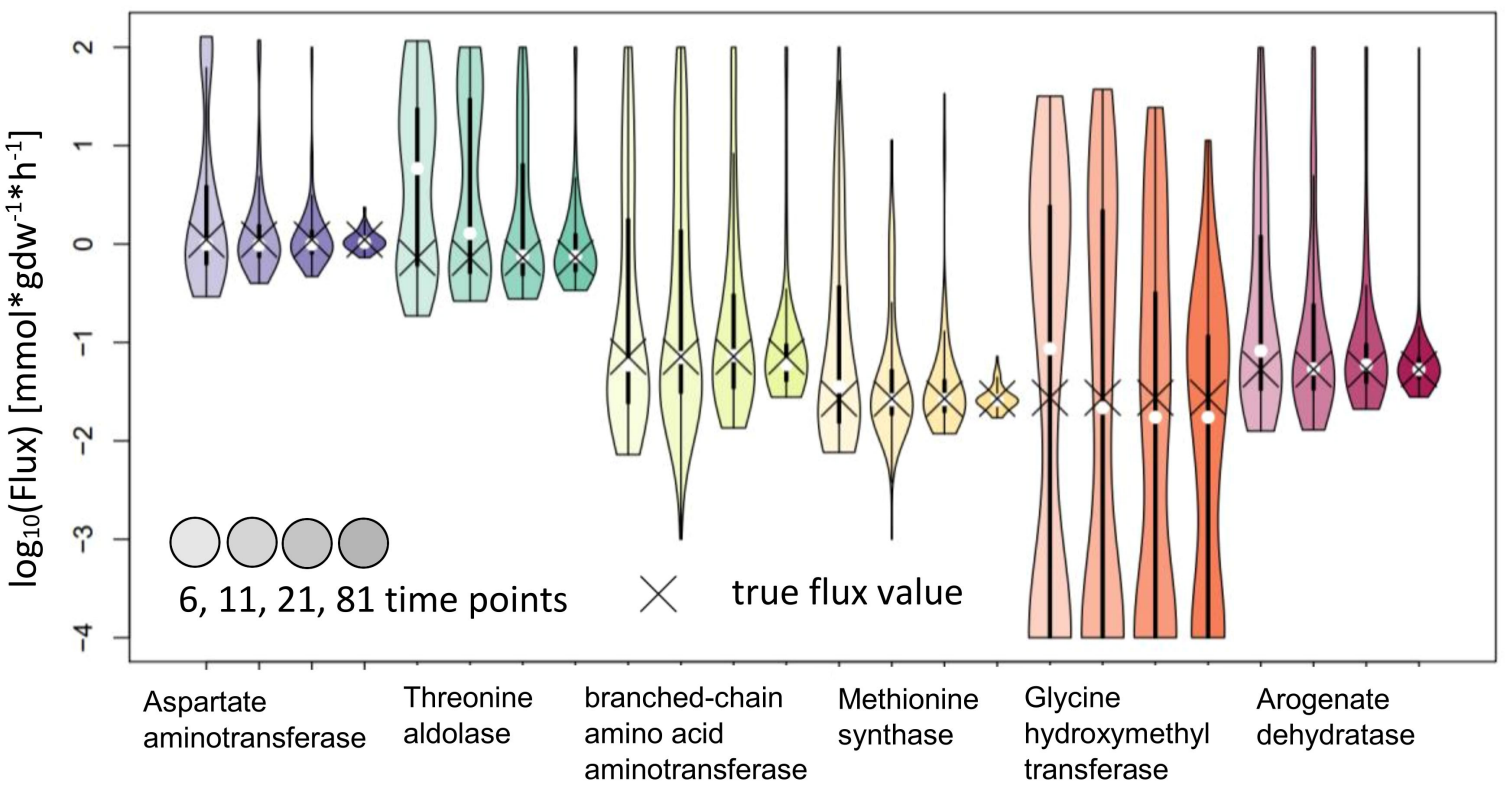
Flux estimates and sensitivity analysis of IsoSim for 6, 11, 21 and 81 time points for AraCore reactions. The confidence intervals of the estimated fluxes of individual reaction from AraCore generally span several orders of magnitude. Only the estimates for flux values for Aspartate aminotransferase and Methionine synthase have confidence intervals of less than one order of magnitude, but this was achieved using the very high number of 81 time points.

IsoSim results in estimates with confidence intervals that include the true value for all reactions tested. While this means that the method is reliable, most of the confidence intervals are very large and, as a result, the actual flux is not precisely estimated. We observed that the usage of 81 time points led to a higher precision for the flux estimates of Aspartate aminotransferase and Methionine synthase, and with confidence intervals whose upper bound is 2.5 to 3-fold larger than the lower bound (**Figure 7**).

**Figure 8:**
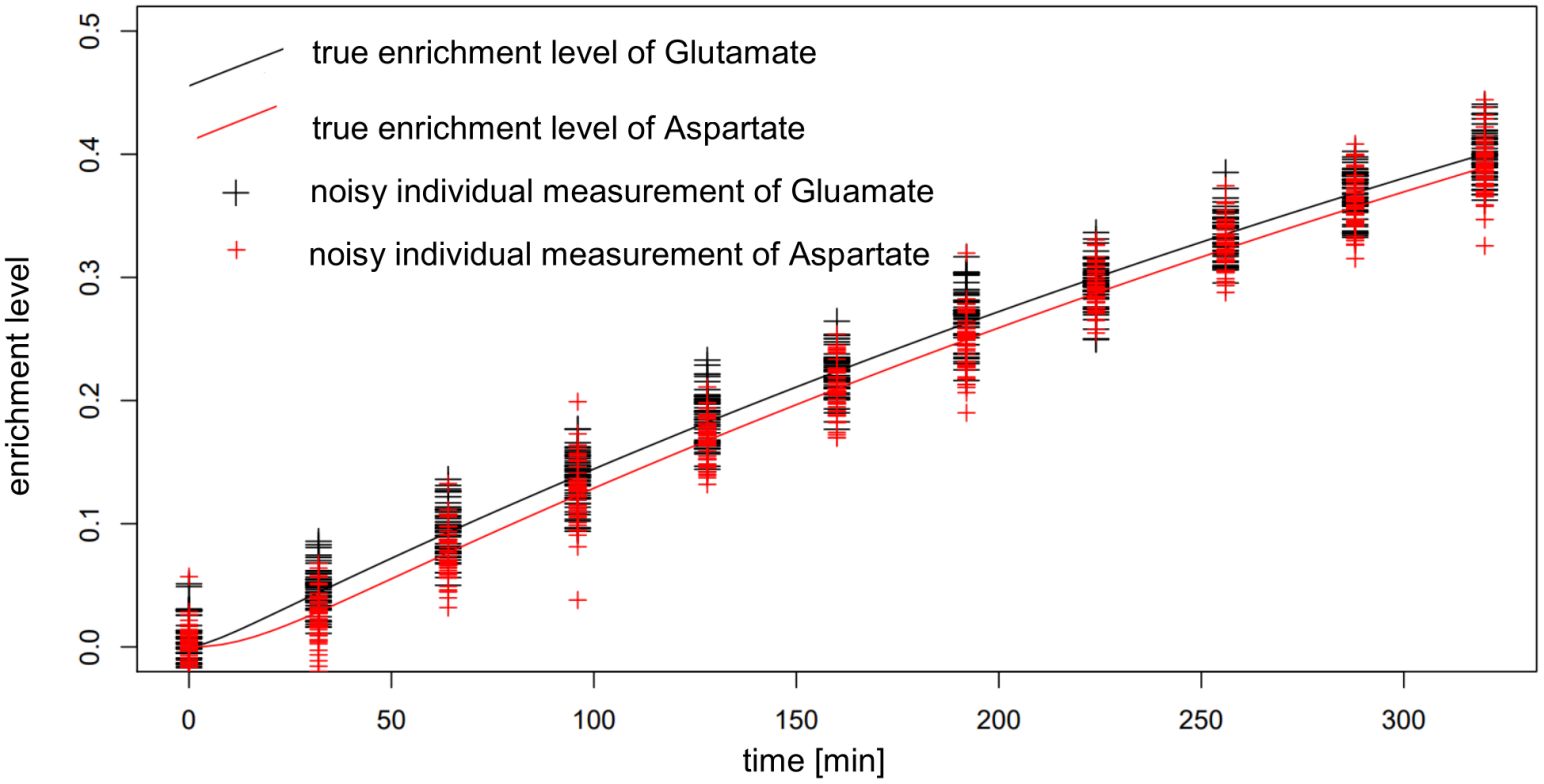
Actual and noisy data for simulated enrichment of Glutamate and Aspartate in AraCore. The distance between the enrichment of Glutamate and Aspartate, being reactant and product of Aspartate aminotransferase, is smaller than the expected random deviation of the measurements.

One factor to be considered in interpreting these findings is the very small difference in the labeling of reactant and product in the reactions whose fluxes are estimated. This difference reaches a maximum of around 0.01, well within the expected standard deviation of the measurements (**Figure 8**), which leads to bad estimates. To overcome this issue, additional measurements with possible supply of another label (e.g. ^13^C) could be employed, if applicable. The consequence of these findings is that IsoSim is applicable for reliable estimate of fluxes of reactions that involve metabolites with a difference of their label enrichment that is bigger than the standard deviation of the enrichment measurements.

**Figure 9:**
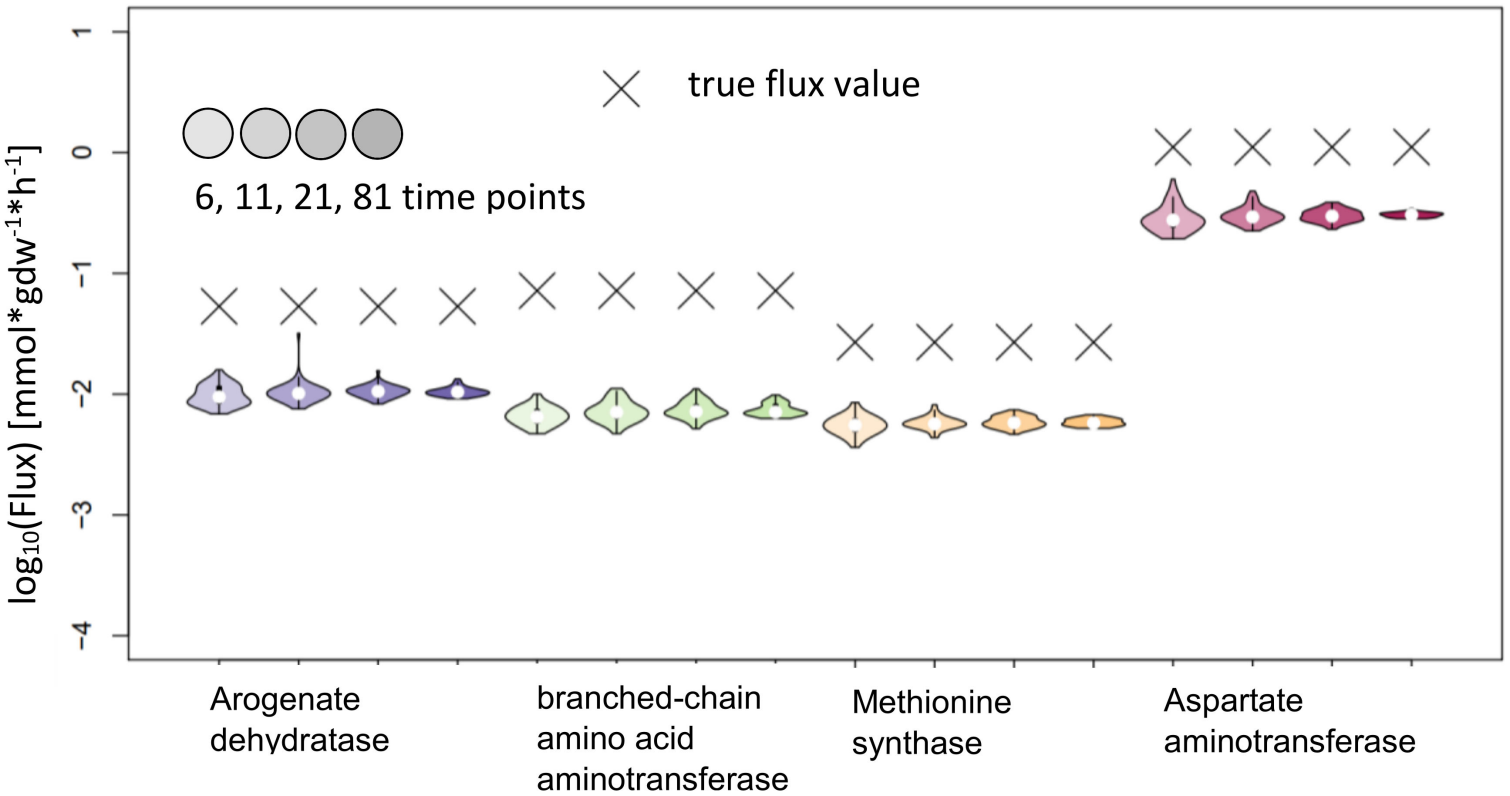
Flux estimates and sensitivity analysis of KFP for 6, 11, 21 and 81 time points for AraCore reactions. The confidence intervals of the estimated fluxes of individual reaction from AraCore are narrow, yet the true flux values are outside of them.

The test of KFP on the reactions from AraCore failed, the confidence intervals are small, but the true values are not contained in these intervals (**Figure 9**). Further analysis showed multiple potential reasons for our findings, indicating that the basic version of KFP may not be suitable in our settings. One factor again is the very small difference of only 0.01 in the labeling of reactant and product in the reactions whose fluxes are to be estimated. This is particularly relevant, as the fit for the labeling of the reactant has an error larger than this difference. Furthermore, the labeling of the metabolites only reached 0.4 in the simulated time span, further complicating the fitting of the input function(s).

**Figure 10:**
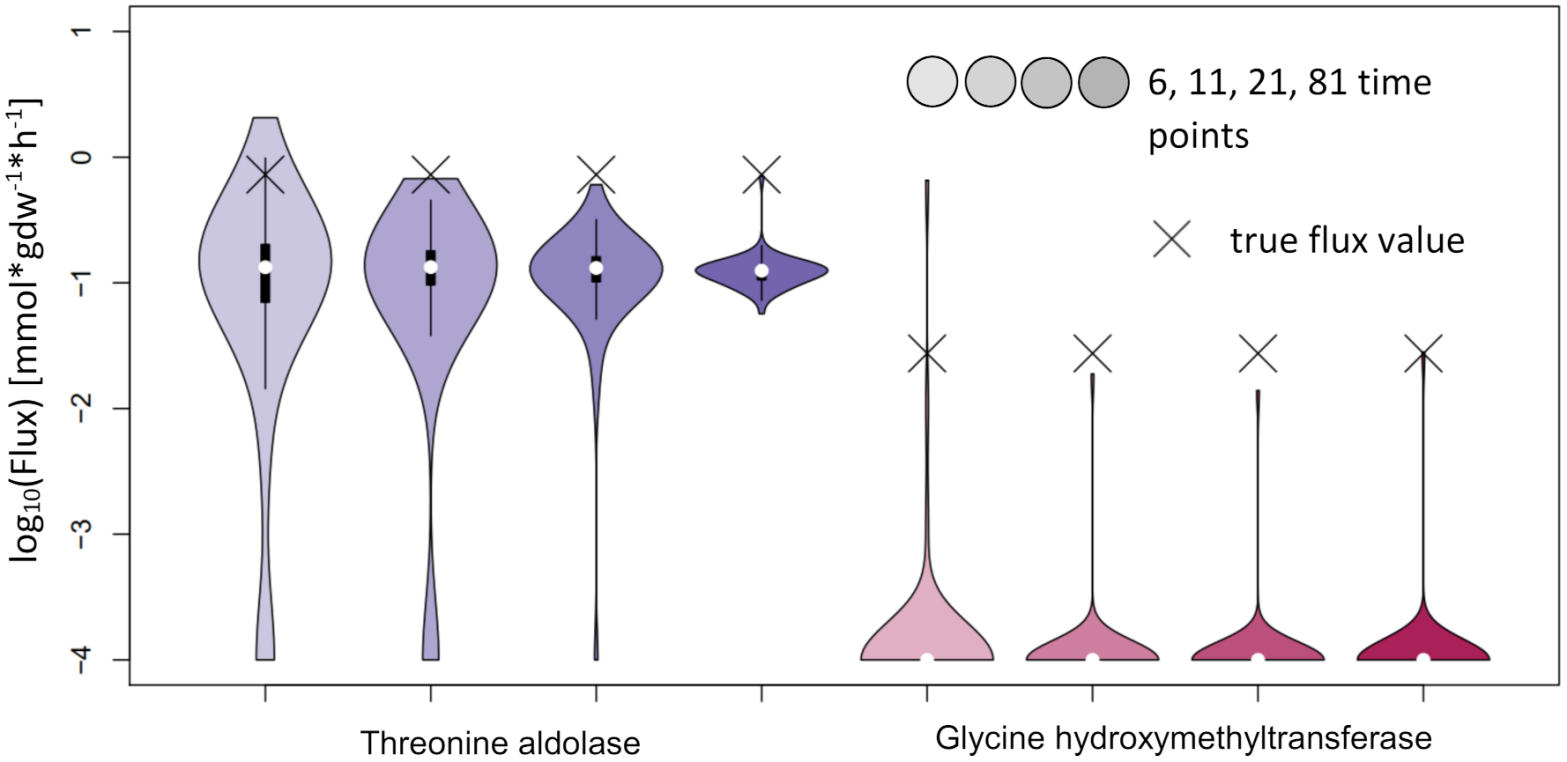
Flux estimates and sensitivity analysis of NSMFRA for 6, 11, 21 and 81 time points for AraCore reactions. The confidence intervals of the estimated fluxes of individual reactions from AraCore are rather big and yet the true flux values are outside of them. The flux value of Glycine hydroxymethyltransferase was mostly estimated to be zero.

For NSMFRA, our implementation showed poor results for the pair of reactions chosen (Threonine aldolase and Glycine hydroxymethyltransferase). For both reactions the confidence interval is rather big and still the true flux values are not covered (**Figure 10**). The flux value of Glycine hydroxymethyltransferase was mostly estimated to be zero although the true value in the simulations was 0.027 mmol*gdw^-1^*h^-1^. The comparative analysis estimates are based on our implementation of the core principles of NSMFRA using the input fitting method of IsoSim. NSMFRA might perform better with their original implementation, which, however, is not publically available.

## 9 Discussion

The goal of this comparison of local INST-MFA approaches for isotopically nonstationary metabolic flux analysis was to evaluate their performance in estimating fluxes of individual reactions. To this end, we used simulated enrichment data for a small synthetic metabolic network and for a large-scale metabolic network model of *A. thaliana*. We note that the cases considered in the simulations are not representative of all metabolic flux analysis, since there are few atoms to be labeled when feeding ^15^N in comparison to ^13^C-labeled nutrients. We used data sets with varying number of time points to investigate the effect that this factor has on the precision and accuracy of flux estimates.

### 9.1 Best performing method

IsoSim was the overall best performing method and is applicable to any part of the metabolic network for which labeling enrichment data of sufficient metabolites are available. We found that its estimates were reliable, although their confidence intervals were not very precise in all cases. Nevertheless, we recommend increasing the number of Monte Carlo runs of the flux estimation for sensitivity analysis significantly, e.g. to 100 instead of the default value of 4. The simpler KFP approach in its basic form is limited to a specific reaction pattern (**Figure 1A**). It showed comparable results for metabolites near the labeled input of the metabolic network but was not applicable in other cases. The addition of a delay parameter could extend the applicability of KFP to reactions further downstream of the metabolic network, as could the usage of other fitting functions for other network constellations (Szecowka et al., 2013). NSMFRA is limited to the network motif of converging alternative pathways (**Figure 1C**) and without the original implementation it is not reliable in difficult settings as the reaction from AraCore, with a very small difference in the enrichment level of reactants and products. The main difference to IsoSim is the ability to calculate the ratio of the two converging reactions without measurement of the absolute concentrations of the involved metabolites.

### 9.2 Impact of network structure

The position of a reaction in the network and the resulting delay of enrichment of its reactants and products, along with the size of the resulting difference in the enrichment labels of reactants and products, have a big impact on the performance of all three methods. IsoSim is able to handle the delay of the enrichment of reactants and metabolites of a reaction rather well, while the basic form of KFP, like NSMFRA, is not applicable with a large delay, as the exponential decay functions used for fitting do not cover this case.

The position in the network has an even stronger effect for reactions in the larger network of AraCore. For the selected reactions of AraCore, IsoSim did not result in accurate flux estimates, in contrast to the findings for the synthetic network. Importantly, all flux estimates were associated very large confidence intervals. NSMFRA and KFP failed to accurately estimate the fluxes of these reactions, and the true values were outside the confidence intervals of the estimates. One common characteristic of all six reactions from AraCore is the very small difference in the labeling between reactants and products (**Figure 8**), which is a likely reason for the observed difficulties in flux estimation.

### 9.3 Recommendations

Our findings demonstrated that the availability of sufficient time points proved essential to achieve flux estimates with small confidence intervals. IsoSim required at least 11 time points for precise flux estimates in the test cases considered. Consequently, flux estimation should be performed with data from experiments that include as large number of time points as possible. The large number of time points at which measurements are taken is necessary to estimate fluxes even for reactions where reactants and products show a larger difference in their labeling. While this leads to increased effort, it does not invalidate the advantage of lower computational complexity of local vs. global INST-MFA approaches. We stress that in the cases where KFP is applicable, it produced estimates with a small confidence interval even with only 6 time points.

One point found in all six reactions from AraCore is the very small difference in the labeling between reactants and products (**Figure 8**), which is one likely reason for the observed difficulties in flux estimation. Our recommendation to address this problem is to select reactions close to the labeled input or to choose reactions where the product has a sufficiently large pool size to slow down its enrichment; this will in turn result in a larger difference of the enrichment levels between the products and the reactants of the reaction.

Further, for all three local approaches, the flux estimates are expected to improve as more of the adjoining metabolic network is integrated in the estimation (Szecowka et al., 2013, Hörl et al., 2013; Millard et al., 2020). One obvious example are fully coupled reactions, as r_3_ and r_7_ in the synthetic example (**Figure 2A**): they are fully coupled and have in steady state the same flux values, yet the estimate of IsoSim for r_3_ (**Figure 4A**) has a much smaller confidence interval than the estimate for r_7_ (**Figure 4B**). Accordingly, the estimate of r_3_ should also be used for r_7_. ScalaFlux/IsoSim can be scaled to include larger sub-networks or even the entire network, as long as the enrichment of all input and output metabolites is measured. The number of measured metabolites thus limits the size of the sub-network. The recommended proximity to the input or to metabolites with large pool sizes can further limit the network selection.

Regarding further developments of local isotopically nonstationary MFA, it is worth noting that for IsoSim the medians of confidence intervals for the flux estimates even for the reactions from AraCore were rather close to the true values. If the flux estimates could be further filtered by yet unused, but valid criteria to exclude unlikely flux values, this will reduce the number of outliers in the estimates; this in turn will improve the confidence intervals by rendering them smaller. A possible candidate for this filtering is the goodness of the individual label input fits. Another option is to identify if for some simple reaction patterns the resulting ODEs can be solved analytically (as in KFP), which is expected to improve the quality of the estimate.

Even if good local estimates are only possible for a small range of reactions in a large network, the inclusion of those local flux estimates as constraints in flux balance analysis (FBA) problems can lead to more pronounced reductions in the solution space of FBA problems due to the imposed reaction couplings.

## Data availability statement

Publicly available datasets were analyzed in this study. This data can be found here: https://github.com/sebahu/Local_INST-MFA.

## Author contributions

Conceptualization, ZN. Implementation, SH. Formal Analysis, SH. Writing – Original Draft, SH, ZN. Writing – Review and Editing, SH and ZN. All authors contributed to the article and approved the submitted version.
